# Self-Selected Leisure Promotes Ambulatory Blood Pressure Dipping: A Within-Person Randomized Field Experiment

**DOI:** 10.3390/bs16010148

**Published:** 2026-01-21

**Authors:** Marcellus M. Merritt, Matthew J. Zawadzki, Jack M. Cowger

**Affiliations:** 1 Department of Psychological and Brain Sciences, University of Wisconsin Milwaukee, Milwaukee, WI 53211, USA; jmcowger@uwm.edu; 2 Department of Psychological Sciences, University of California Merced, Merced, CA 95343, USA; mzawadzki@ucmerced.edu

**Keywords:** leisure, stress management, blood pressure, dipping, cardiovascular, obesity

## Abstract

An early indicator of future cardiovascular risk is lower levels of nighttime blood pressure (BP) dipping from day to night. Prior work has been limited to identifying health behaviors that can promote greater dipping. This pilot study proposes that one possible set of behaviors may be engagement in self-selected leisure activities (SSLAs, or freely chosen non-work activities that are performed with the purpose of relaxation and/or mental escape), which have been linked with reduced daily stress and general daily BP control. Healthy young adult college students [*N* = 32; 78.1% (*n* = 25) female, 71.9% (*n* = 23) white, with an average body mass index (BMI) of 26.31 (SD = 2.46)] visited our laboratory twice within approximately one week. At each visit, the participants were fitted with an ambulatory monitor to collect BP over 24 h. On each day, participants were randomly assigned to either engage in an agreed-upon SSLA or go about their day as usual, except to refrain from engaging in assigned SSLAs; compliance was verified by daily diaries. When accounting for BMI and race/ethnicity, the results showed a higher percentage of BP dipping on the SSLA versus control day for diastolic BP (d = 0.54). SSLAs may be associated with reduced future cardiovascular disease through a nighttime BP dipping effect.

## 1. Introduction

In healthy adults, blood pressure (BP) typically decreases when one shifts from wake to sleep, otherwise known as BP dipping. Non-dipping is an early indicator of future cardiovascular disease risk and mortality (Emlek & Aydin, 2022; Fedecostante et al., 2015; Kulecki et al., 2025; Lempiäinen et al., 2024; Viera et al., 2012). Moreover, the magnitude of diastolic and systolic BP dipping inversely predicts mortality (Fedecostante et al., 2015; Lempiäinen et al., 2024; Sakhuja et al., 2019).

Although low dipping and non-dipping are known risk factors, identifying the factors associated with dipping is essential for effective interventions. Some studies have suggested that commonly explored health behaviors, including physical activity, smoking, alcohol intake, and obesity (e.g., body mass index), are modestly related to dipping levels (Memioğlu et al., 2025; Viera et al., 2012). However, the significant levels of unexplained variance in these models suggest that other factors account for the dipping levels. Indeed, psychological stress has been identified as a factor that predicts poor levels of BP dipping (Barksdale et al., 2013; Birmingham et al., 2023; Linden et al., 2009; Spruill, 2010). Moreover, interventions aimed at reducing stress resulted in improved BP dipping (Sherwood et al., 2017). Although promising, this work was conducted with participants in whom coronary diseases are already present, thus limiting our understanding of the potential of using stress management techniques as a proactive measure. Moreover, this stress management intervention used time- and resource-intensive approaches, limiting its potential reach and adoption.

Engagement in flexible stress-coping strategies may provide a solution to these issues. One promising strategy is engagement in self-selected leisure activities (SSLAs) or freely chosen non-work activities that are performed with the purpose of relaxation and/or mental escape (Ahn, 2025; Bian & Xiang, 2023; Lee et al., 2024; Merritt et al., 2017; Sezer & Aki, 2024). Leisure has shown the potential to reduce stress and induce positive mood (Ahn, 2025; Elsden et al., 2022; Fancourt et al., 2021; Tepeköylü-Öztürk & Soytürk, 2025; Zawadzki et al., 2015, 2021). Moreover, those who engage in more SSLAs have healthier cardiovascular profiles than those who engage in fewer SSLAs, including lower resting BP levels (Mausbach et al., 2017), and lower ambulatory BP levels (Zawadzki et al., 2013). Yet, it is unclear whether SSLAs can modulate the independent risk factors of BP dipping.

The purpose of the current study was to test the novel hypothesis that engagement in SSLAs is linked with more nighttime BP dipping. We enrolled young adults using a within-person field experiment comparing days when SSLAs were assigned to be performed to non-SSLA days. The within-person field experiment design allowed each participant to serve as their own control, thereby isolating day-to-day variation in BP associated with SSLA engagement while holding stable individual differences constant. By focusing on contrasts within the same individual, the design reduces confounding by time-invariant factors such as sex, baseline health, trait stress, or habitual BP levels. Moreover, as comparisons are made on similar days about a week apart, many potential day-level confounds (e.g., typical caffeine intake, sleep habits, or work demands) are unlikely to differ systematically between SSLA and non-SSLA days.

Demonstrating potential benefits at a young age is a conservative test of our hypothesis, as BP levels are typically more controlled and normotensive in young adults, including with more BP dipping (i.e., Bello et al., 2020; Forshaw et al., 2022; Palatini et al., 2020). Yet, demonstrating effects with young adults is important as a preventative strategy as behavioral patterns solidify as one enters an emerging adulthood period, and these patterns can predict future morbidity and mortality before early signs of chronic disease manifest (Garett et al., 2017). Significant effects of completing leisure during the day and the resultant BP dipping that night would demonstrate the potential of SSLAs as a more user-friendly and preventative coping strategy that could help offset future cardiovascular diseases (Duerden, 2022; Zoeteweij et al., 1991).

## 2. Materials and Methods

### 2.1. Participants

For extra credit points (as approved by the university human subjects review board), 362 college students completed a short Qualtrics screening survey to assess eligibility for the study, including a BMI of 18 or higher and no personal or family history of major medical conditions such as high BP, diabetes, or stroke. These factors jointly ensure a healthy sample, but who may have early risk factors for future diseases and could benefit from preventative interventions. Eligibility also included typical engagement in leisure activities that could be performed while wearing an ambulatory BP monitor (e.g., no swimming or intense physical activity that could damage the monitor). The tactic of offering students one extra credit hour to complete the survey and two to complete each 24-h session of ambulatory BP monitoring is an accepted standard in recent American Psychological Association ethical principles of human research, as it is a nominal incentive that historically has not had undue or coercive effects on participation in surveys and psychophysiological research (American Psychological Association, 2003).

**Figure 1:**
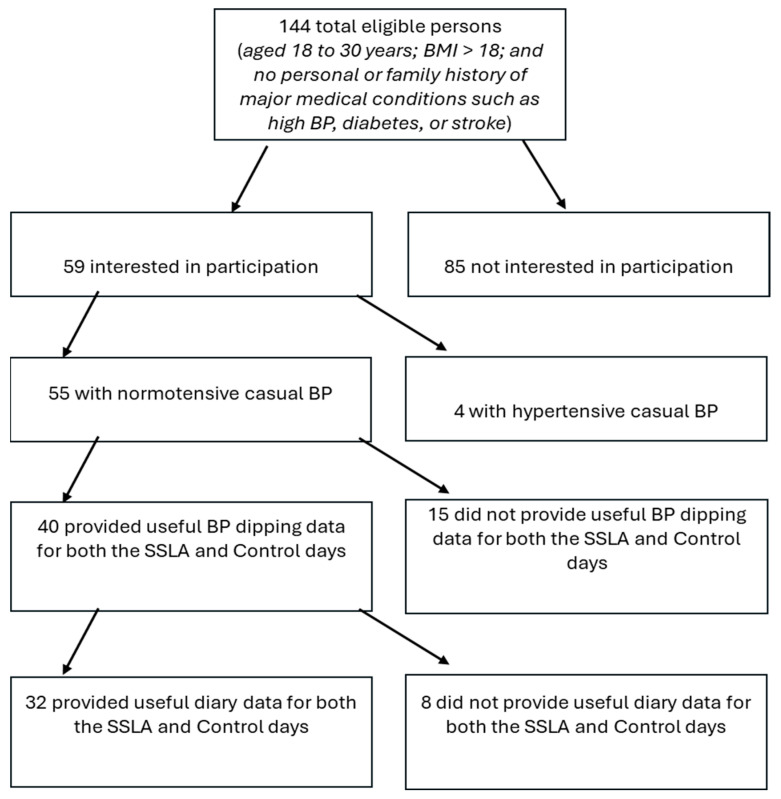
A detailed progression (CONSORT chart) of participants from screening to inclusion in final analysis.

A total of 144 people were eligible (most participants were ineligible due to low BMI scores) with 85 of these people not interested in participating, not having ample time for the lab visits, or not showing up for lab visits (*n* = 14) (see Figure 1 for a detailed progression of participants from screening to who was included for final analysis). The remaining 59 participants were initially enrolled to complete the protocol. Based on in-lab BP readings, four participants had readings in the hypertensive range (i.e., ≥140/90 mmHg) and thus were excluded. Out of the remaining 55 subjects, 40 (73%) provided useful BP dipping data for both the SSLA and control days. Out of the remaining 40 subjects, 32 (80%) provided useful diary data for both the SSLA and control days. The final sample consisted of 32 adults, aged 18–31 years (mean = 20.8 years), 78.1% (*n* = 25) female, 71.9% (*n* = 23) white, 12.5% (*n* = 4) African American, and 15.7% other (including Hispanic American and Middle Eastern), with an average BMI of 26.31 (SD = 2.46). Assuming a medium effect size (d = 0.50) with acceptable power (0.75) to detect the effect at the *p* < 0.05 level, G*Power (v. 3.1.9.4) analyses (Faul et al., 2007) suggested that for a two-tailed t-test comparing means for matched pairs (SSLA vs. non-SSLA within-person), a total sample size of 30 was acceptable.

### 2.2. Materials

Online survey. After providing informed consent via an online waiver, participants completed online baseline surveys, with basic sociodemographic items such as self-identified racial/ethnic group membership, a brief health history (e.g., self-reports of height in inches and weight in pounds), and a 27-item SSLA questionnaire (see Merritt et al., 2017). The latter asked for brief descriptions of the three most typical activities that a participant did to unwind and/or take them mentally away from the most stressful aspects of their life. For each activity, we asked about the frequency, duration, and subjective qualities (e.g., relaxation or challenge).

SSLA operational definition. During the first laboratory visit, we conducted newly created 10-min semi-structured interviews to assess the usual SSLAs and narrow them down to one SSLA to be performed in the field. For participants, we defined leisure as anything one does in their free time away from work, school, domestic, or other obligations that is/are done to get away, relieve stress, bring enjoyment, re-energize, or regroup. Thus, it could be just about any kind of activity, such as work-related tasks, physical activity, social interactions, or almost any private pursuit. This interview verified the three most typical activities that a participant completed to unwind and/or take them mentally away from the most stressful aspects of their life. To narrow down to one SSLA, each participant was asked to describe each activity in terms of frequency and usual duration in the last month, which activity they preferred the most, and which were the easiest to do, among other questions. The experimenter worked with the participant to select the activity based on activities that were most preferable and frequent, ethically feasible (e.g., listening to music vs. smoking marijuana), and most easily. Thus, the agreed-upon SSLA could have been any activity that allowed for reliable assessments of ambulatory BP (see Table 1) but was not illegal or criminal in nature.

Ambulatory blood pressure measurement. Ambulo 2400 ABP (Mortara Instrument, Inc., Milwaukee, WI, USA) was used to measure ambulatory BP. The Association for the Advancement of Medical Instrumentation (AAMI) standards have been confirmed as a reliable and accurate assessment of ambulatory BP (Alpert, 2010). A BP reading was obtained every 30 min from each participant during self-reported wake times and hourly during sleep times. Measurements were averaged over the sleep period and over the wake period (sleep and wake times validated using a sleep diary). To be included, participants had to have at least five wake and three nighttime BP readings during each measurement session to ensure sufficient reliability in wake and sleep averages (Barnes et al., 2008; Llabre et al., 1988). To calculate dipping, we took the difference of day BP from night BP and divided this by day BP; this measure was calculated as the percentage of dipping at night from daytime measurements, which was calculated separately for systolic BP and diastolic BP (Loredo et al., 2004).

Daily diaries. We confirmed leisure compliance on the SSLA day and leisure non-compliance on the control day by asking subjects to complete four self-initiated paper and pencil diaries on each day at uniform timepoints (10:00 a.m., 2:00 p.m., 6:00 p.m., and 10:00 p.m.). Each diary included an open-ended item about engagement in any favored leisure activity since the last diary entry that was used to track compliance. Other diary items not relevant to the present manuscript included occurrence of relevant health behaviors since the last diary entry (e.g., exercise and caffeine), ratings of moods and cognitions at the time of the dairy entry, and any interpersonal interactions in the previous 30 min. SSLA absorption (“Please rate how absorbed you were in the activity”) and relaxation (“How relaxing was the activity”) were self-reported items in each diary on a scale from 0 ‘Not at All’ to 6 ‘Very Much.’ The score for each item was taken from the diary entry in which an SSLA was reported. Each diary overall took about 3 min on average to complete. We decided to track compliance verification by way of self-reported diaries at fixed times mainly since it allowed us to collect a uniform range of assessment points per subject.

### 2.3. Procedure

Eligible participants visited a psychophysiology lab twice, approximately a week apart. At the first visit, after providing informed consent, participants completed the SSLA interview, and then two seated resting BP measurements were taken with an Omron 3 series BP monitor (BP7100; Omron Healthcare, Inc., Lake Forest, IL, USA) to validate the non-hypertensive status. At both visits, participants were fitted with a validated Ambulo 2400 monitor to measure BP over the following 24 h. Once fitted, sample readings were taken to familiarize the participants with the Ambulo 2400 assessment protocol. Participants were then told to go about their day as they would normally while sleeping with the monitor on, with the only exceptions being refraining from water-based activities (e.g., showering or swimming) or other physically strenuous activities (e.g., weightlifting or aerobics) that may impede the reliability of the Ambulo 2400 readings.

Finally, participants were randomly assigned when to engage in their allocated SSLA to counterbalance which type of day was the first for each participant. On an ‘SSLA day’, the participants were instructed to engage in the agreed-upon SSLA at the time of their choice before going to bed. On the control day, participants were instructed to go about their day as usual, except for asking them to refrain from engaging in any favored leisure activity that day (i.e., the researcher verbally advised the participant to refrain from engaging in the agreed-upon leisure activity or any other favored leisure activity). So, we were able to use the diaries to exclude any subjects from related analyses who reported either not performing the assigned SSLA on the SSLA day or performing the assigned or other SSLA on the control day (see Figure 1 for the inclusion of useful diary entries).

## 3. Results

Assessment of SSLA compliance. All statistical tests were run using SPSS version 31 software. About 87.5% (i.e., 35 out of 40 fully counterbalanced BP dipping participants) reported performing the favored activity on the SSLA day and 89.7% (i.e., 35 out of 39 fully counterbalanced BP dipping participants) reported not performing a favored activity on the control day. Since some favored activities are unavoidable (e.g., watching TV or interacting socially with friends), a few participants performed them on the control day, reducing the overall sample size by a modest amount. Lastly, 91.4% (i.e., 32 out of 35) of the remaining participants completed both the SSLA and control day diaries. Overall, we obtained good compliance with the agreed-upon SSLAs, with a caveat being that some activities such as watching TV or socializing are uniquely difficult to refrain from.

Watching TV or programming online was the most reported leisure activity, followed by listening to music and reading (Table 1). All participants participated in agreed-upon leisure activities that would be referred to as passive (i.e., not physically strenuous activities). Overall, participants had daytime BP levels in the normotensive range: systolic BP (Control: M = 121.18, SD = 9.68; SSLA: 123.70, SD = 12.48) and diastolic BP (Control: M = 74.16, SD = 6.18; SSLA: 77.19, SD = 6.92). In addition, participants had nighttime BP levels in the normotensive range: systolic BP (Control: M = 105.96, SD = 13.75; SSLA: 104.68, SD = 15.31) and diastolic BP (Control: M = 66.42, SD = 7.57; SSLA: 65.68, SD = 8.17).

SSLA engagement and BP dipping. Paired samples *t*-tests were run to compare average daytime and nighttime BP levels on an SSLA day to their control day. The results showed a higher average diastolic BP level on SSLA days versus control days for diastolic BP (t(31) = −2.40; *p* = 0.022; Cohen’s d = 0.44). There were no other significant effects for nighttime diastolic BP (t(31) = 0.597; *p* = 0.555) or systolic BP during daytime (t(31) = −1.27; *p* = 0.215) or nighttime (t(31) = 0.539; *p* = 0.594).

**Figure 2:**
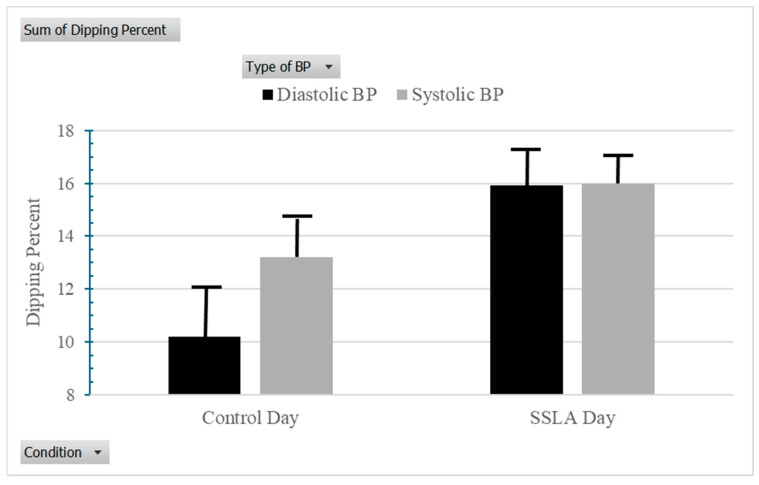
Ambulatory blood pressure dipping (in percentage) on control day versus SSLA day. Note. BP = Blood Pressure; SSLA = Self-Selected Leisure Activity. Error bars represent pooled standard errors for each mean score.

As a test of hypotheses, paired samples *t*-tests were run to compare BP dipping on an SSLA day to their control day. Supporting the predictions, the results showed more BP dipping on SSLA days versus control days for diastolic BP (M = 15.92, SD = 9.12 vs. M = 9.83, SD = 10.13; t(31) = −2.90; *p* < 0.007; Cohen’s d = 0.54) (see Figure 2). This association was marginally significant for systolic BP (M = 15.47, SD = 7.39 vs. M = 12.75, SD = 8.53; t(31) = −1.86; *p* = 0.073; Cohen’s d = 0.34).

Finally, to test for the potential covariates of race/ethnicity and BMI in the SSLA and diastolic BP dipping link, a hierarchical regression model (HMR) was run with the predictor variables entered in the following order to predict SSLA day diastolic BP dipping: (1) control day diastolic BP dipping and (2) BMI and race/ethnicity. This model allowed us to assess if SSLA day diastolic BP dipping was different from control day diastolic BP dipping alone and then when accounting for these covariates. Obesity status was included as a covariate given that obesity is a major early risk factor for future high BP, stroke, and cardiovascular diseases (Bastien et al., 2014; Memioğlu et al., 2025; Papathanasiou et al., 2015). Also, African Americans (versus comparable White Americans) show a higher risk in daily BP control (Dua et al., 2014; Memioğlu et al., 2025).

In step 1 [Fchange (1, 30) = 8.11; *p* < 0.008], the results confirmed that control day diastolic BP dipping is significantly different from SSLA day diastolic BP dipping [t(30) = 2.85; *p* < 0.008]. Step 2 was not significant [Fchange (2, 28) = 0.72; *p* < 0.496]; however, the relationship between control day diastolic BP dipping and SSLA day diastolic BP dipping remained significant [t(28) = 2.69; *p* < 0.012]. Neither BMI [t(28) = −1.18; *p* < 0.247] nor race/ethnicity [t(28) = 0.34; *p* < 0.734] was significantly associated with SSLA day diastolic BP dipping.

Exploratory tests. We explored differences in BP dipping levels based on the type of SSLA performed. Scores for SSLA absorption and relaxation were taken from the diary entry in which an SSLA was reported. If the agreed-upon SSLA was reported twice, then the average of these ratings was calculated. Days in which a participant rated their SSLA as more absorbing were inversely correlated with same-day diastolic BP dipping (r(42) = −0.312; *p* = 0.044). However, the correlation between ratings of relaxation while engaged in the assigned SSLA and same-day diastolic BP dipping was not significant (r(41) = −0.126; *p* = 0.434). Ratings of absorption were positively correlated with ratings of relaxation (r(41) = 0.558; *p* = 0.001).

## 4. Discussion

An early sign of future cardiovascular risk is low BP dipping from day to night (Lempiäinen et al., 2024; Sakhuja et al., 2019; Viera et al., 2012). Identifying when low levels of dipping and non-dipping occur and intervening to promote BP dipping has the potential to prevent future cardiovascular disease and mortality. As expected, we found in a sample of young adults that there was more nighttime BP dipping on a day on which one was randomly assigned to complete an SSLA than on another day when one was assigned to not complete an SSLA. We observed these effects across a wide range of SSLAs, including some activities that are more passive in nature, suggesting that the benefits of SSLAs in improving BP dipping are multifaceted.

These results present a strong counterpoint to recent research indicating that commonly studied health-related behaviors, including physical activity and smoking, are unrelated to BP dipping (Fedecostante et al., 2015; Kulecki et al., 2025; Sakhuja et al., 2019). Nevertheless, future work should examine if some types of SSLAs are more effective in improving dipping than others, as well as testing possible mechanisms for how SSLAs confer their benefits. In addition, the average daytime diastolic BP levels were marginally higher on the SSLA day than on the control day. This result may be a function of extra attention and physical effort to engage in agreed-upon leisure activity on the SSLA day. Yet, the levels of physical activity may be only one component of many that is important. We did not see significant correlations between ratings of the perceived relaxation levels (a possible inverse of physical activity) of the SSLA and BP dipping, but higher self-reported mental absorption while engaged in the assigned SSLA was associated with less diastolic BP dipping on that day. This absorption might have been an indicator of participants trying to distract themselves from stressors. This explanation is in line with other work suggesting that absorption in leisure was only beneficial for low levels of rumination (Zawadzki et al., 2013). Thus, future studies should account for key psychosocial mechanisms and barriers for related SSLA engagement without actively requesting participants to do so.

Prior work has demonstrated that leisure reduces stress and improves mood (Merritt et al., 2017; Fancourt et al., 2021; Zawadzki et al., 2015), promotes better consistency and/or predictability in daily activity patterns (Carney et al., 2006; Monk et al., 2003), and enhances the sense of control and coping, which under certain circumstances have shown benefits to BP (Merritt et al., 2004, 2017; Silvia et al., 2011). Discerning which of these pathways and others are yet unidentified will be critical to designing an intervention focused on optimal engagement in leisure activities (Atefi et al., 2025).

Still, future research should look at possible health disparities for active versus passive leisure activities systematically (e.g., Tepeköylü-Öztürk & Soytürk, 2025). For instance, does jogging as a favored (vs. platonic) leisure activity result in more BP dipping than a more passive activity (e.g., novel reading) deemed favored (vs. platonic)? Yet, the fact that we obtain powerful trends in BP dipping for less active or passive SSLAs is uniquely impressive.

In line with this, it is also important to examine alternative explanations, such as how SSLAs might interact with other health behaviors (e.g., physical activity levels and nighttime sleep quality) to modulate daily cardiovascular responses. Other factors independent of and/or interactive with a stress reduction mechanism could be at play such as variation in evening routines, anticipation of the leisure activity, or differences in behavioral activation linked to specific activities which might also add to changes in nocturnal BP. Future research should aim to examine the generalizability of the effects, testing the impact of SSLAs on individuals with more crystallized daily routines (e.g., intense physical activity regimes), who have an early risk of cardiovascular disease (e.g., diabetes or stroke), and/or have higher degrees of obesity status.

Also, we acknowledge that the definition of leisure time activity was ad hoc and, as a result, diverse. It could include activities as diverse as jogging and smoking marijuana, which themselves have clear effects on BP. However, as subjects refrained from high-risk and physically strenuous activities (e.g., weightlifting, aerobics) that may impede the reliability of the Ambulo 2400 readings, we believe that those activities were not likely confounding factors. Moreover, it was vital to determine how a desired leisure time activity was selected for each participant to perform or avoid. That is, the leisure activity is already part of the subject’s daily repertoire and hence should have a corresponding health benefit not seen in uniformly assigned behavioral interventions. Overall, favored leisure is broad but despite this range, there is an effect.

In some cases, like the systolic BP dipping effect, there were smaller effects than anticipated. Yet, it is conceivable that persons in the control group engaged in leisure on these days. If so, this would produce a more conservative test as the control group may be obtaining the presumed beneficial component of leisure if someone did not follow instructions. That said, participants did not report engaging in their selected leisure on a non-SSLA day in the daily diary. It is possible that participants were falsely reporting compliance or avoided one type of SSLA but performed others. Future work may benefit from validating these self-reports against objective measures (e.g., Fitbit assessments for physically oriented activities) and more precise self-reports of health behaviors (e.g., frequency and amount of daily caffeine usage). Yet, given the broad nature of SSLAs, objective or optimal validation of certain favored activities is not logistically possible without significant intrusion (e.g., meditation practice or TV watching).

Another future direction is to evaluate if the utility of assigning leisure was rate-limited by obesity status. This is vital because, in general, overweight and obese individuals have worse dipping profiles than those at healthy weights. Then, we would have a viable tool that may help this vulnerable group. Future studies need to determine why this may be the case (e.g., how daily leisure activity modulates parasympathetic stress mechanisms linked with obesity and daily BP control) (Blalock et al., 2025; Hojo et al., 1997; Kulecki et al., 2025).

## 5. Conclusions

In conclusion, this within-person field experiment offers initial evidence that engaging in a self-selected leisure activity during the day is associated with greater nighttime BP dipping, particularly for diastolic BP, in a young, largely normotensive sample. These findings extend prior work on stress, leisure, and cardiovascular regulation by identifying a potentially modifiable, low-cost, and user-friendly behavioral pathway that may influence an early marker of future cardiovascular risk before clinical disease emerges. Although effect sizes were modest and several methodological limitations warrant caution, the consistency of the diastolic dipping effect across diverse leisure activities is notable. Taken together, the results suggest that encouraging individuals to intentionally engage in favored leisure activities may represent a feasible preventative strategy for promoting healthier circadian BP regulation. Larger, more diverse longitudinal studies are now needed to clarify mechanisms, boundary conditions, and long-term clinical significance, but the current findings underscore the promise of leisure not as a trivial pastime but as a meaningful component of cardiovascular health promotion.

## Figures and Tables

**Table 1:** Description and prevalence of SSLAs performed by participants.

SSLA	Number of Subjects	Percent of Sample
Watch TV or online media	13	40.63
Listen to music	4	12.50
Read	4	12.50
Play video games	3	9.38
Nap or sleep	1	3.12
Sing or play instrument	1	3.12
Socialize	1	3.12
Other (i.e., to-do list, be alone)	5	15.63

## Data Availability

The data supporting the findings of this study are available on request from the corresponding author, Marcellus M. Merritt. The data are not publicly available because they contain information that can compromise the privacy of the research participants.

## Abbreviations

The following abbreviations are used in this manuscript:

BMI	body mass index
BP	blood pressure
SSLA	self-selected leisure activity
